# Efficient adoptive transfer of autologous modified B cells: a new humanized platform mouse model for testing B cells reprogramming therapies

**DOI:** 10.1007/s00262-021-03101-4

**Published:** 2021-11-08

**Authors:** Audrey Page, Emilie Laurent, Didier Nègre, Caroline Costa, Véronique Pierre, Thierry Defrance, François-Loïc Cosset, Floriane Fusil

**Affiliations:** CIRI – Centre International de Recherche en Infectiologie, Univ Lyon, Université Claude Bernard Lyon 1, Inserm, U1111, CNRS, UMR5308, ENS Lyon, 46 allée d’Italie, 69007 Lyon, France

**Keywords:** Humanized immune system, B cell adoptive transfer, Cell therapy, Gene therapy

## Abstract

Here, we report a novel experimental setup to perform adoptive transfer of gene-edited B cells using humanized immune system mice by infusing autologous HIS mouse-derived human B cells “educated” in a murine context and thus rendered tolerant to the host. The present approach presents two advantages over the conventional humanized PBMC mouse models: (i) it circumvents the risk of xenogeneic graft-versus-host reaction and (ii) it mimics more closely human immune responses, thus favoring clinical translation. We show that the frequencies and numbers of transduced B cells in recipient’s spleens one week post-transfer are within the range of the size of the pre-immune B cell population specific for a given protein antigen in the mouse. They are also compatible with the B cell numbers required to elicit a sizeable immune response upon immunization. Altogether, our findings pave the way for future studies aiming at assessing therapeutic interventions involving B cell reprogramming for instance by an antibody transgene in a “humanized” hematopoietic setting.

**Supplementary Information:**

The online version contains supplementary material available at 10.1007/s00262-021-03101-4.

## Introduction

B cells are key players of adaptive immunity, not only because they produce antibodies, but also owing to their status of antigen-presenting cells and to their immunoregulation capacity via cytokine production. The development of gene-edited B cells has become increasingly considered for the treatment of a wide range of diseases [1]. Among the many applications of genetically modified B cells, two have appeared as particularly promising in terms of therapeutic intervention. The first one relates to hematopoietic stem cell transplantation (allo-HSCT). One of the main drawbacks of HSCT is the delayed immune reconstitution, especially of the B cell compartment, which takes several months (up to 1–2 years) and results in increased susceptibility of the patients to opportunistic bacterial, fungal and, most importantly, viral infections. Recently, the concomitant injection of allogenic B cells and HSCs has been used to counteract immune defects and to rapidly confer humoral immune protection to transplanted patients [2]. The second promising therapeutic approach concerns the reinfusion of reprogramed autologous B cells to confer protective immunity without vaccination. It relies on viral vectors that can deliver transgenes encoding neutralizing antibodies in host-derived B lymphocytes. This procedure is superior to so-called passive immunization inasmuch as it enables patients to produce therapeutic antibodies for a longer, if not for life-long periods as compared to the injection of short-lasting humanized antibodies. Moreover, the use of reprogrammed B cells for therapeutic purposes is not limited to antibody production since B cells have also been genetically modified to produce regulatory cytokines [3], tolerogenic molecules [4], or non-immune therapeutic proteins such as factor VIII [3, 5]. Overall, the reinfusion of autologous B cells that have been reprogrammed beforehand would be particularly beneficial for the treatment of chronic infectious diseases [6–8], cancers [9, 10] or autoimmune diseases [11]. The term “instructive immunotherapy” has been coined to describe the immediate and robust immune protection achieved by infusion of genetically reprogrammed immune effector cells. Last but not least, a good manufacturing practices (GMP) protocol was developed in 2017 to allow a novel B cell-based strategy aiming to support humoral antiviral immune responses based on adoptive transfer of autologous memory B cells [2, 12].

Several immunocompetent or transgenic mouse models are already being used to study such immunotherapies; yet, there is an unmet need for humanized in vivo models that can more closely mimic human immune responses or improve safety before clinical translation. Here, we report a novel experimental setup to perform adoptive transfer of transduced B cells using humanized immune system (HIS) mice by infusing autologous HIS mouse-derived human B cells “educated” in a murine context and thus rendered tolerant to the host. Despite its relative complexity, this model will prove useful for testing clinically relevant therapeutic strategies and for addressing fundamental questions regarding the immunological consequences of reinfusing reprogrammed B cells to the host.

## Methods

### Lentiviral vector production and titration

Lentiviral vectors (LVs) encoding the green fluorescent protein (GFP) under the B cell-specific FEEK promoter [13] were generated by transient transfection of 293 T cells through calcium phosphate precipitation as previously described [14].

### Mouse experiments

Immunodeficient NOD Scid^–/–^γ_c_^–/–^ (NSG) mice were housed under specific pathogen-free conditions. Experiments were carried out in accordance with the European Union and French National Committee recommendations, under agreement APAFIS#9827–2,017,031,516,233,716 v3. Young NSG (4–5 weeks old) were humanized for the hematopoietic system by intravenous (*i.v.*) retro-orbital injection of 1 × 10^5^ HSCs (CD34^+^ cord blood cells, HIS mice) under anesthesia. Human CD34^+^ cells were purified by positive selection from cord blood obtained from the Lyon Sud Hospital (Lyon, France) upon informed consent or were purchased from Lymphobank. Mice were conditioned by [15] intraperitoneal injection of busulfex (20 mg/kg) 36 h before HSC injection. Infused CD34^+^ cells were pre-activated in vitro by a cytokine cocktail of Flt3 (100 ng/ml), SCF (100 ng/ml) and TPO (30 ng/ml) during 24 h prior to infusion [14]. Blood samples were harvested every 3 weeks starting from 8 weeks post-humanization, to follow the kinetics of humanization by flow cytometry. Animals above > 40% humanization were enrolled for further adoptive transfer experiments. Human immune reconstitution levels were determined using the following calculation method: humanization level = % human CD45^+^ cells/(% human CD45^+^ cells + % murine CD45^+^ cells).

For in vitro transduction, human CD19^+^ B-cells were isolated from donor spleens by magnetic positive selection (CD19^+^ isolation) and pre-stimulated during 16–20 h with cross-linked hCD40L (2 µg/ml), hIL-4 (2 ng/ml) and hBAFF (10 ng/ml) in StemMACS medium. They were then transduced for 6 h with protamine sulfate (8 µg/ml) and a GFP-encoding lentiviral vector used at a multiplicity of infection (MOI) of 10 to 20. B cells were then washed twice in PBS before *i.v.* injection into the retro-orbital sinus of HIS recipient mice under isoflurane anesthesia. Each mouse received up 1 × 10^6^ transduced B cells. Mice were sacrificed 7 days post-adoptive transfer for blood and spleen collection and analysis. In parallel, 1 × 10^5^ cells were kept in culture for three days to perform FACS analysis of GFP-positive cells.

### Flow cytometry

Frequencies of human hematopoietic cells in humanized mice blood or splenocytes were determined with a cocktail of antibodies directed against mouse CD45-VioBlue, huCD3-APC, huCD19-PE-Vio770, huCD20-PE-Vio770, huCD45-VioGreen, hCD27-VioGreen, hIgD-VioBlue and hIgM-APC (all from Miltenyi Biotec). Briefly, cells were resuspended in PBS containing 2% FCS and incubated with an optimal dilution of fluorochrome-conjugated antibodies for 30 min after FcR blocking (Miltenyi Biotec) before being washed twice in PBS containing 2% FCS. Data were acquired on the FACSCanto-II (BD Biosciences) and analyzed with the FlowLogic™ software.

### Statistical analysis

All data were analyzed with GraphPad Prism 8 (Graph-Pad Software).

### Combined Results and Discussion

**Fig. 1:**
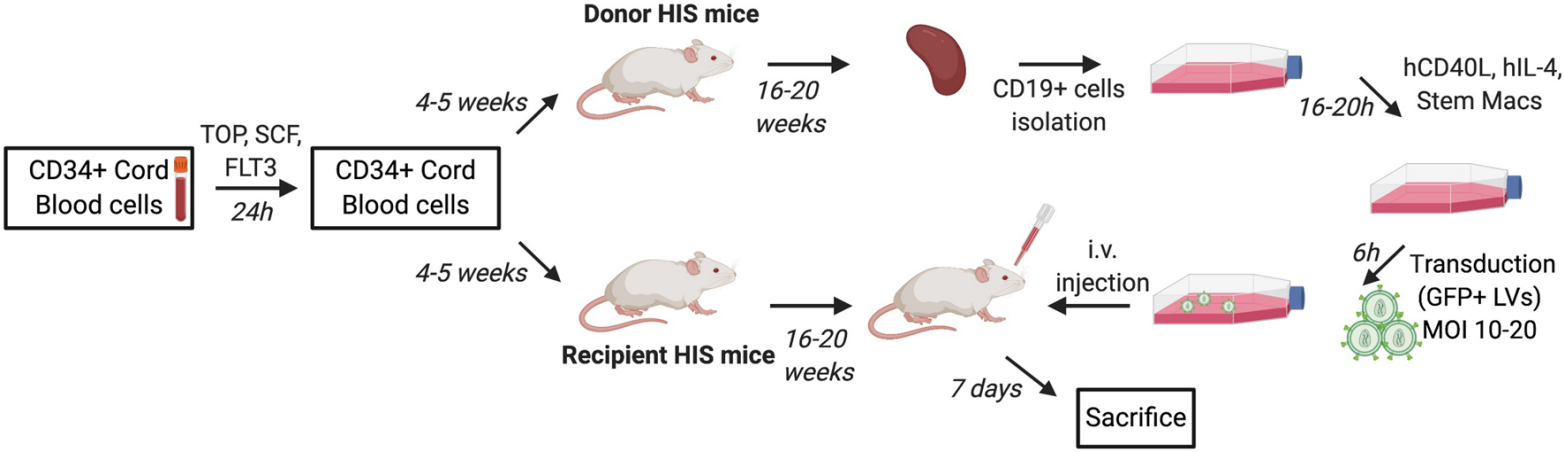
Set-up for adoptive transfer of modified B cells in HIS mice. Young NSG mice (4–5 weeks) were infused with pre-activated CD34 + cord blood cells. The humanization score was followed by flow cytometry for 16–20 weeks. B cells were isolated from the spleens of HIS donor mice displaying a humanization score above 40% for huCD45^+^ cells and superior to 5% for T cells. B cells were activated during 16 to 20 h prior to lentiviral transduction. Six hours after transduction, modified B cells were injected intravenously in recipient “autologous” HIS mice (*i.e.,* humanized with the same source of CD34+ cells as donor HIS mice). Recipient mice were sacrificed one week after cell infusion and the ratios of GFP+ cells were analyzed by flow cytometry in the spleen

We aimed at developing an efficient protocol for adoptive transfer of autologous modified B cells using HIS mice (Fig. 1). For this purpose, 1 × 10^6^ B cells isolated from splenocytes of humanized mice were transduced with a BAEV GP (Baboon endogenous virus envelope glycoprotein)-pseudotyped lentiviral vector encoding GFP and subsequently infused by *i.v.* route in recipient autologous HIS mice. Spleens of donor mice (8 to 15 donor mice were used depending on the cohort) were pooled and submitted to positive selection with anti-huCD19 Abs. Between 4 × 10^6^ to 5 × 10^8^ human B cells were obtained after selection depending on both the cohort and the humanization rate of donor mice (Sup Fig. 1a–c). B cell purity after magnetic sorting ranged between 81 and 93%. It has previously been published that the human immune system in the peripheral blood is mainly composed of B cells until 10–14 weeks and that T cells start to reach the periphery at this time [16]. As expected, at this late stage of humanization (> 20 weeks post-humanization), we detected more than 80% of human cells in the spleen, mostly T cells (> 60% CD3^+^ cells), except for the cohort #C for which the humanization rate was lower (Sup Fig. 2). As previously described for other humanized mouse models [17], most splenic B cells exhibited a naïve phenotype (CD20^+^ CD27^−^ IgM^+^ IgD^+^) (Sup Fig. 3).

**Fig. 2:**
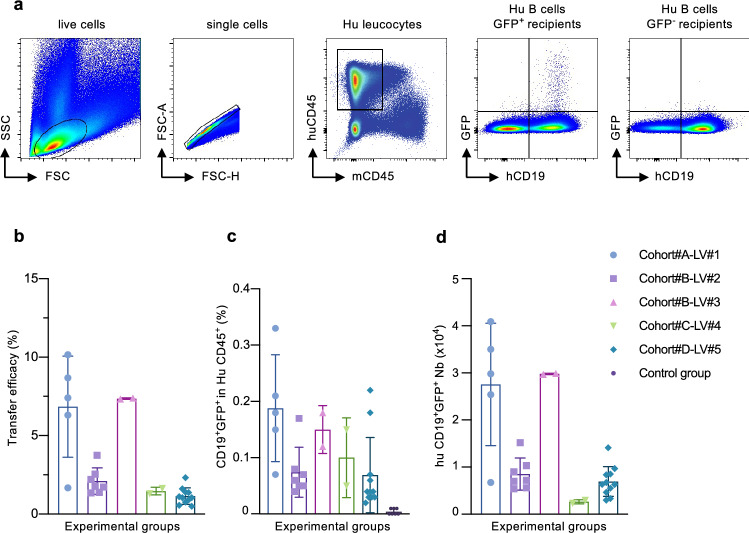
Efficacy of the adoptive transfer of engineered B cells in HIS mice. Four cohorts of NSG mice were humanized with 4 different batches of huCD34^+^ cells. B cells were isolated from donor mice and injected into autologous HIS recipients after lentiviral transduction (Cohort #A (n = 5), #B (n = 9), #C (n = 2), #D (n = 11)). Five different LV batches were used for B cell transduction: LV #1 (n = 5), LV #2 (n = 7), LV #3 (n = 2), LV #4 (n = 2), LV #5 (n = 11). The control group was performed with non-transduced B cells (n = 7). (**a**) Gating strategy. Representative plots are presented. (**b**) Adoptive transfer (AT) efficacy calculated as the ratio of the numbers of infused GFP^+^ B cells to the numbers of GFP^+^ splenic B cells post-transfer. (**c**) Frequencies of huCD19^+^GFP^+^ cells among huCD45^+^ splenocytes in recipient HIS mice analyzed by flow cytometry 7 days after B cell transfer. (**d**) Absolute numbers of huCD19^+^GFP^+^ B cells in recipient’s spleens

Our previous results showed that the transduction efficiency of human B cell with such pseudotyped viral particles is up to 50% [5], which is comparable to the efficiencies observed here (Sup Fig. 1d–e). One week after adoptive transfer (AT), we analyzed GFP-transduced B cells in the spleens of recipient mice using the gating strategy described in Fig. 2a. The phenotype of recovered B cells after AT is very similar of donor B cells, suggesting that the activation/transduction step as well as the transfer does not affect the B cell phenotype since donor B cells and recipient splenic cells present the same markers (Sup Fig. 3). No GFP^+^ B cells were detected in the peripheral blood of these mice (Sup Fig. 4). The AT efficiency in these experiments was calculated as the ratio between the number of splenic GFP^+^ B cells one week after transfer and the number of GFP^+^ B cells initially injected in the recipient. As illustrated by Fig. 2b, the AT efficiency ratio ranged from 1.2 to 10% (Fig. 2b). We observed differences of AT efficiency between each group, which could be explained by the source of B cells (*i.e.*, the source of HSCs used for humanization, cohort #A, #B, #C or #D), by the batch of LVs (cohort #B, LV#2 and cohort #B LV#3, same donor but 2 different LV batch) or by a potential differential impact of the pre-transduction activation procedure on subsequent B cell engraftment. As a point of comparison, this AT efficiency is within the range of the recovery rate of BCR transgenic mouse B cells following their adoptive transfer performed in immunocompetent recipients with a polyclonal or a quasi-monoclonal B cell repertoire [18]. Importantly, the rate of post-transfer recovery of transduced B cells is also comparable to that described for adoptive transfer of human T cells isolated from HIS mice into autologous HIS mice [19]. As shown in Fig. 2c, GFP-expressing cells accounted for 0.04–0.22% of human B cells in recipient’s spleens. By way of comparison, the frequency of B cells reactive against the (4-hydroxy-3-nitrophenyl)acetyl (NP) hapten in naïve immunocompetent mice has been estimated to be around 0.025% [20]. Based on the total number of splenocytes, we estimated the absolute numbers of modified B cells to be between 3 × 10^3^ and 3 × 10^4^ GFP-positive cells (Fig. 2d). These figures are within the range of the size of the pre-immune B cell population specific for Phycoerythrin in the lymph nodes and spleen of naïve C57Bl/6 mice (4 × 10^3^ to 2 × 10^4^) as reported previously [21]. More importantly, in the classically used model of adoptive transfer of monoclonal NP-reactive B1-8 transgenic B cells into AM 14 HEL-reactive BCR transgenic recipients, 1 × 10^4^ Ag-specific B cells are sufficient to give rise to a *bona fide* humoral anti-NP response after antigenic stimulation. In the present study, more than 80% of the infused recipient mice contained 1 × 10^4^ or more GFP-expressing B cells. This suggests that if HIS-derived B cells are reprogrammed with a BCR transgene instead of GFP, the numbers of genetically modified B cells recovered post-adoptive transfer in our present HIS mice model would be sufficient to initiate a sizeable immune response against the targeted Ag. Future experiments should address the issue as to whether reprogrammed B cells can integrate the memory B cell and long-lived plasma cell compartments, thus ensuring long-term production of therapeutic Abs.

To conclude, we propose that humanized mice can be used as a suitable preclinical model to assess therapeutic interventions involving B cell reprogramming in a “humanized” hematopoietic setting. Such approaches are of particular interest for the treatment of many immune-mediated diseases, among which infections, allergies, autoimmune pathologies or cancers. One of the key issues will be to determine whether reprogrammed B cells can integrate the memory B cell and plasma cell compartments thereby ensuring long-term production of therapeutic molecules.

### Supplementary Information

Below is the link to the electronic supplementary material.

Supplementary file1 (PDF 1133 kb)
