# Supplementary material for: Efficient adoptive transfer of autologous modified B cells: a new humanized platform mouse model for testing B cells reprogramming therapies
Source: Cancer Immunol Immunother. 2021 Nov 8;71(7):1771–5. doi: 10.1007/s00262-021-03101-4 (PMC9188505; doi:10.1007/s00262-021-03101-4)
Supplement: Supplementary file 1 — Supplementary file1 (PDF 1133 kb) [file 262_2021_3101_MOESM1_ESM.pdf]

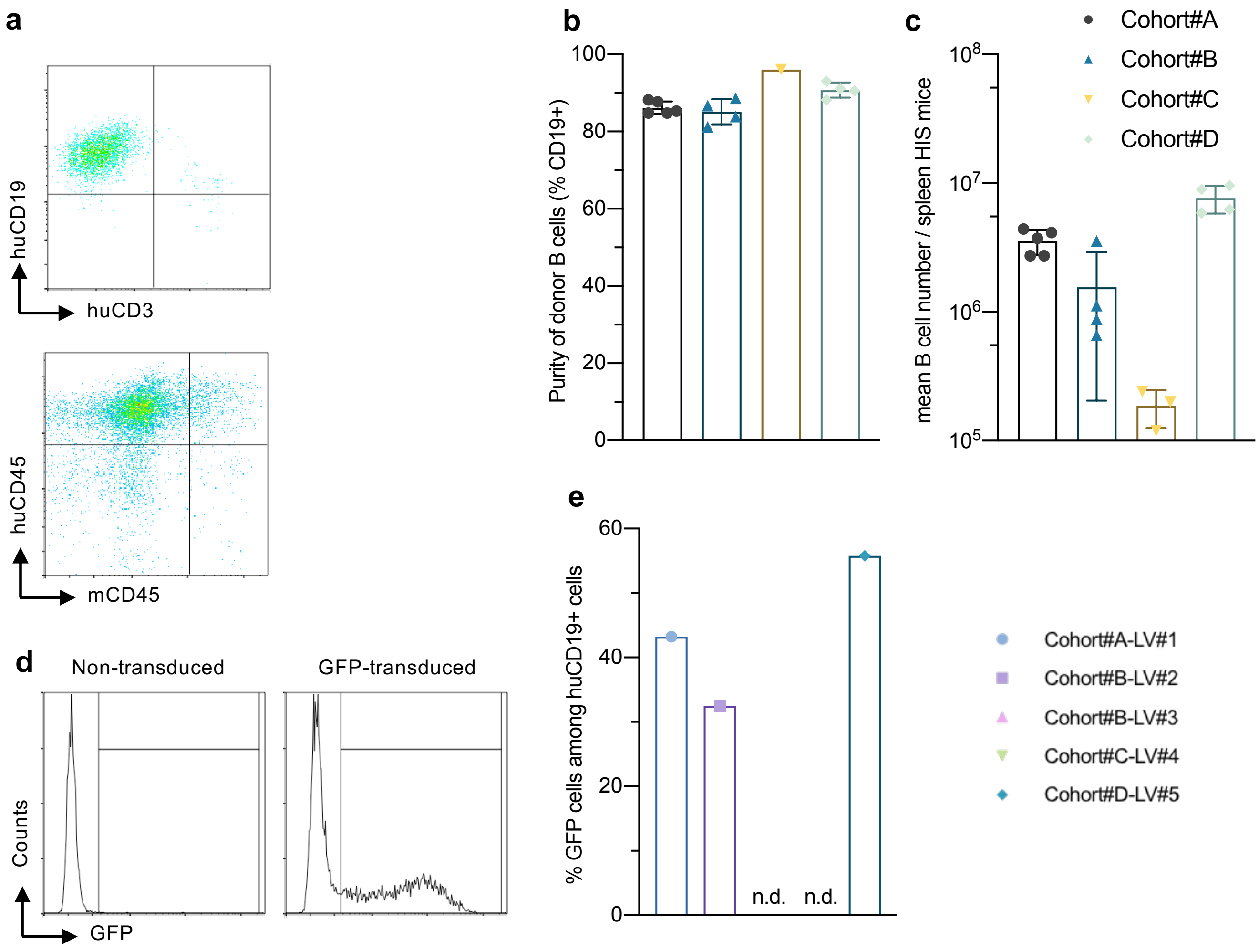

Sup Figure 1. Phenotypical characterization of donor B cells before adoptive transfer. Four cohorts of NSG mice were humanized with 4 different batches of $huCD34^{+}$ cells. B cells were isolated from 8 to 16 donor mice depending on the cohort. B cells were isolated from pooled donor-matched splenocytes and transduced with GFP-encoding lentiviral vectors (LV) before adoptive transfer into recipient mice. (**a**) B cell purity after magnetic huCD19 positive selection of donor splenocytes as assessed by flow cytometry. Representative plots are presented. (**b**) Purity of isolated donor B cells for each cohort. (**c**) Absolute numbers of B cells retrieved from a single donor HIS mice after magnetic hu CD19 positive selection for each cohort. **(d)** Transduction efficiency. For some experiments, a small fraction of transduced B cells were kept in culture for 3 days to assess transduction efficiency by flow cytometry. Representative plots of GFP expression are presented (within the $CD19^{+}$ gate). (**e**) Transduction efficiency evaluated as the percentage of GFP^{+} B cells (n.d., not done).

Cohort#A: pool of 16 donor mice; Cohort#B: pool of 8 donor mice, Cohort#C: pool of 15 donor mice, Cohort#D: pool of 15 donor mice.

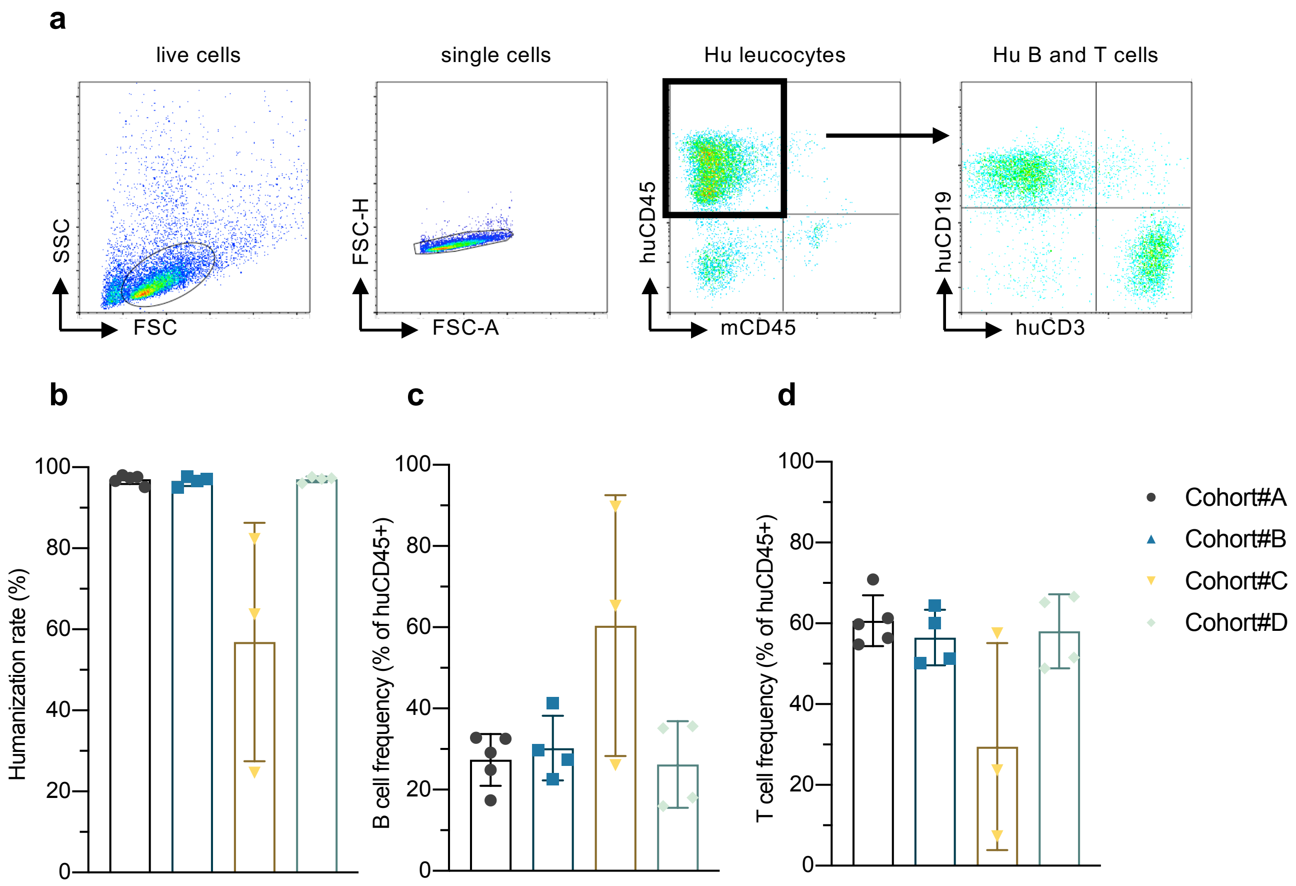

Sup Figure 2. Generation of Humanized Immune System mice. Four cohorts of NSG mice were humanized with 4 different batches of $huCD34^{+}$ cells. For adoptive transfer, donor B cells were isolated by $CD19^{+}$ positive selection from a pool of matched donor splenocytes. (**a**) Gating strategy for analysis of human leucocytes. Representative plots are presented. (**b**) Humanization "rate" calculated as the $huCD45^{+}/(huCD45^{+} + mCD45^{+})$ cell ratios. **(c)** and **(d)** Frequencies of human B ($CD20^{+}$) and T cells ($CD3^{+}$), respectively, among $huCD45^{+}$ splenocytes in donor HIS-mice spleen analyzed by flow cytometry. Cohort#A: pool of 16 donor mice; Cohort#B: pool of 8 donor mice, Cohort#C: pool of 15 donor mice, Cohort#D: pool of 15 donor mice.

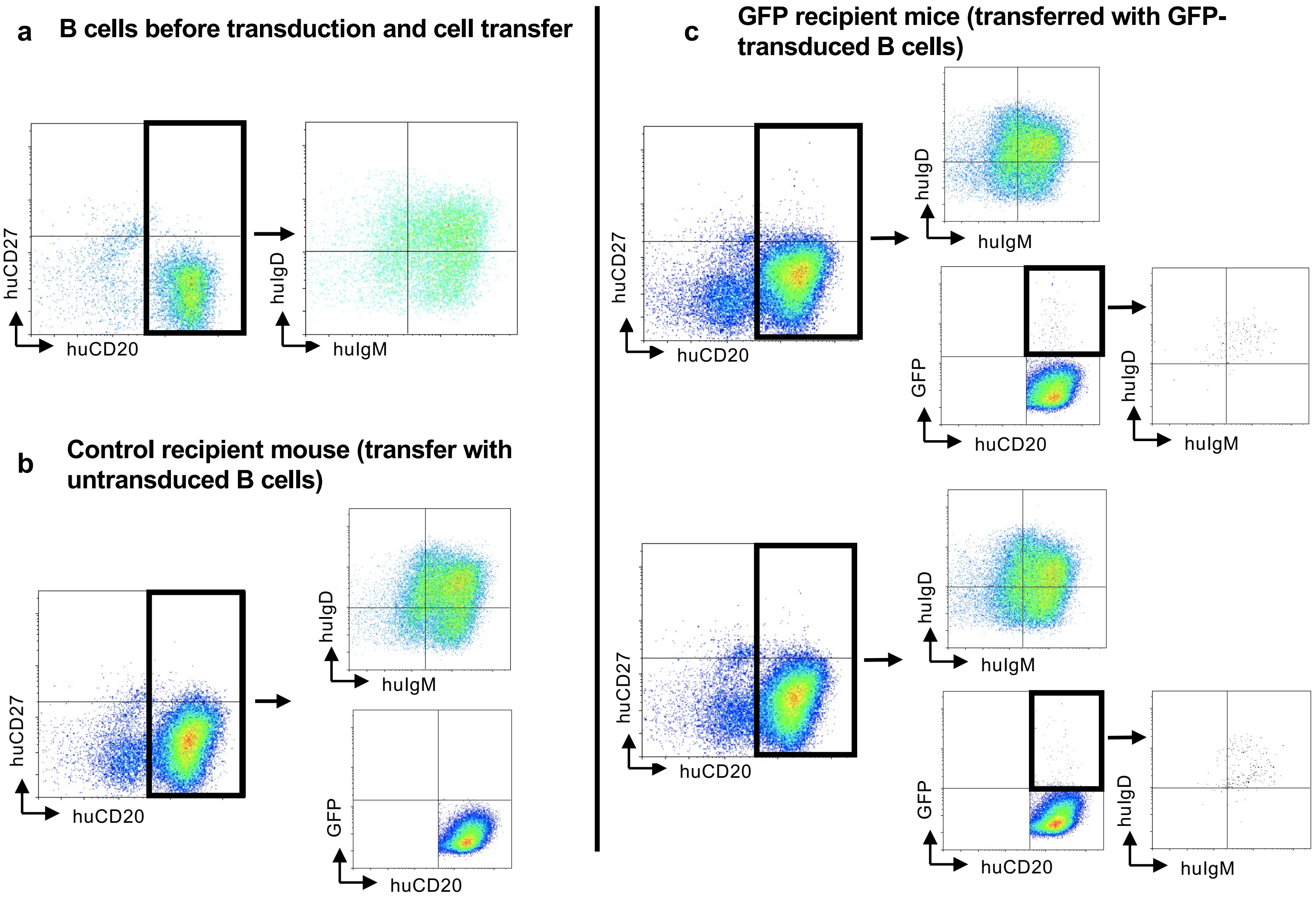

Sup Figure 3. Phenotype of human B cells before and after adoptive transfer. NSG mice were humanized with huCD34^{+} cells. B cells were isolated from donor mice and transduced (or not) with GFP-encoding lentiviral vectors before adoptive transfer in recipient mice. B cell phenotype (CD20, CD27, IgM, IgD) was assessed by flow cytometry before transduction (**a**) and in the spleen of recipient mice (recipient mice were inoculated with non-transduced B cells (**b**) or GFP-transduced B cells (two replicates are showed to show the reproducibility of the data) (**c**).

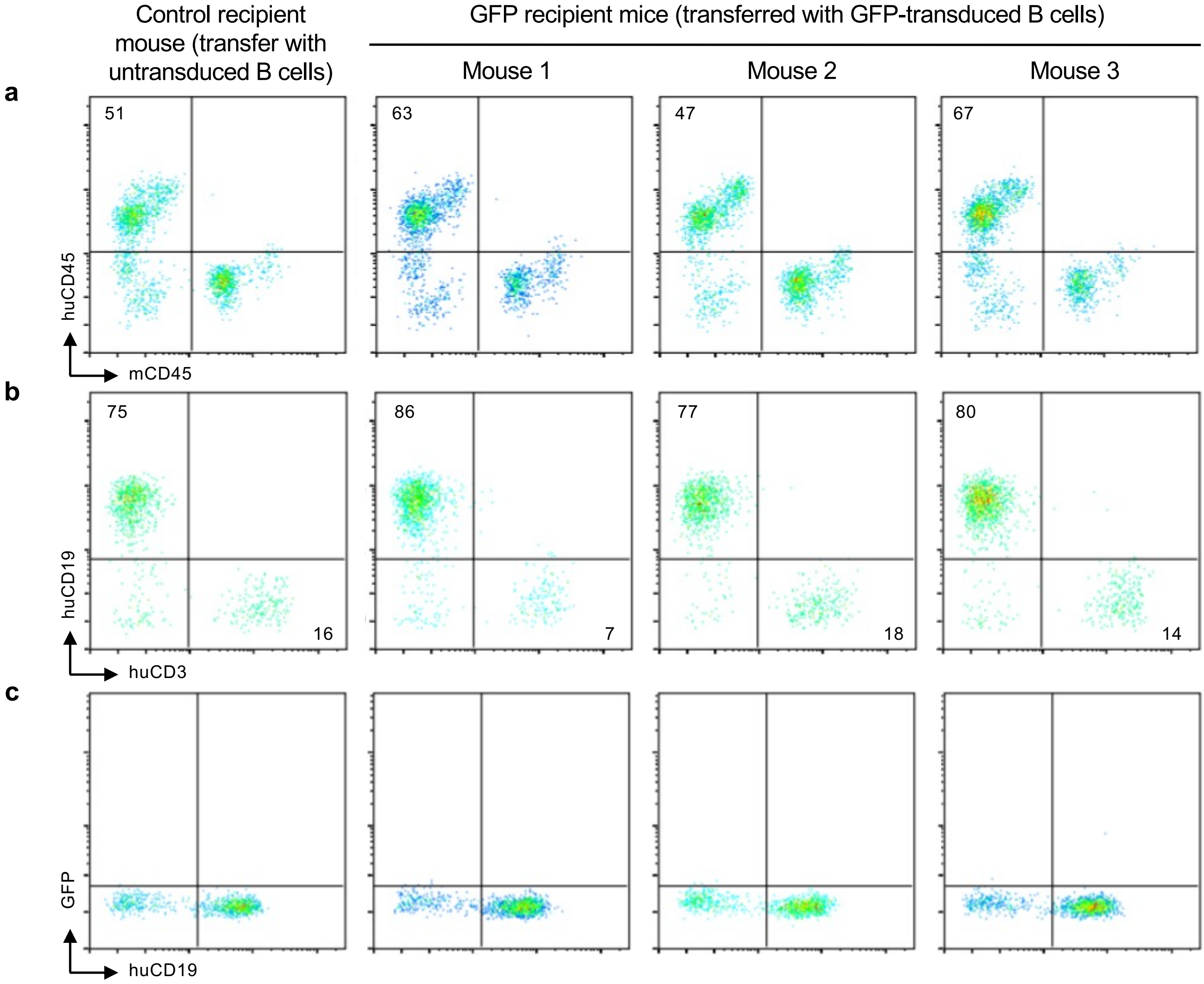

Sup Figure 4. Analysis of the peripheral "human" hematopoietic compartment of HIS recipients after adoptive transfer of GFP-transduced B cells. B cells were isolated from HIS donor mice and transferred into autologous HIS recipients after lentiviral vector transduction with a GFP transgene. In the control group, non-transduced B cells were transferred to autologous HIS recipients. (**a**) Frequencies of huCD45^{+} cells in recipient HIS-mice blood analyzed by flow cytometry 7 days after B cell transfer. (**b**) Frequencies of B (CD19^{+}CD3^{-}) and T (CD19^{-}CD3^{+}) cells among circulating huCD45^{+} cells (huCD45^{+}mCD45^{-} gate) in recipient HIS-mice 7 days after B cell transfer (**c**) Pattern of GFP expression in circulating human leucocytes (huCD45^{+}mCD45^{-} gate) from recipient HIS-mice 7 days after B cell transfer.
